# The psychological impact of COVID-19 on frontline doctors in Tshwane public hospitals

**DOI:** 10.4102/safp.v65i1.5807

**Published:** 2023-12-22

**Authors:** Juliet D. Duffton, Marthinus J. Heystek, Andreas Engelbrecht, Suma Rajan, Renier A. du Toit

**Affiliations:** 1 Department of Family Medicine, Faculty of Health Sciences, University of Pretoria, Pretoria, South Africa; 2 Department of Family Medicine, Division of Emergency Medicine, Faculty of Health Sciences, University of Pretoria, Pretoria, South Africa; 3 Military Health Research Centre, Military Psychological Institute, Pretoria, South Africa

**Keywords:** burnout, depression, anxiety, stress, somatic symptoms, psychological, COVID-19, doctors, frontline, South Africa, pandemic

## Abstract

**Background:**

The coronavirus disease 2019 (COVID-19) pandemic placed immense pressure on frontline doctors. Burnout is a psychological syndrome that develops in response to chronic work stress. It consists of emotional exhaustion (EE), depersonalisation (DP) and reduced personal accomplishment (PA). Burnout is associated with personal dysfunction and compromises the work profession and patient safety. International studies suggest burnout is exacerbated during a pandemic.

**Methods:**

We conducted a descriptive cross-sectional observational study. Respondents included frontline doctors working in emergency medicine, family medicine and internal medicine during COVID-19 in Tshwane public hospitals. The survey included two validated questionnaires, the Maslach Burnout Inventory and the Depression, Anxiety, Stress Scale-21. The aim was to determine the prevalence and severity of burnout, psychological and somatic symptoms in frontline doctors.

**Results:**

Of the 163 participants, we found clinical burnout to be present in 58.9% (*n* = 96) and extreme burnout in 19.6% (*n* = 32). Moderate to extremely severe levels of stress, anxiety and depression were present in 55.1% (*n* = 90), 43.6% (*n* = 71) and 22.1% (*n* = 36) of participants, respectively. We found significant correlations between burnout and psychological symptoms. Increased levels of burnout, anxiety, depression and stress were found to be meaningfully associated with adverse somatic symptoms.

**Conclusion:**

Our study demonstrated an insufferably high prevalence of burnout and psychosomatic symptoms in frontline doctors during COVID-19. In the event of future pandemics, more measures should be taken to support frontline doctors.

**Contribution:**

Pandemic-associated burnout and its psychophysical consequences have not been studied in frontline doctors in South Africa.

## Introduction

Doctors working on the frontlines during the coronavirus disease 2019 (COVID-19) pandemic have endured highly stressful circumstances. Outbreaks of infectious diseases are notorious for their psychological strain on frontline healthcare workers (HCWs).^1,2,3,4,5^ During the severe acute respiratory syndrome (SARS), Middle East respiratory syndrome (MERS) and Ebola outbreaks, studies showed that frontline workers experienced significant stress levels, concerns about the health of their families and themselves, as well as extreme fear, anxiety, societal stigma and prejudice.^2,3,4,5,6^

Burnout among doctors is well described in the literature. It is defined as ‘a psychological syndrome of emotional exhaustion (EE), depersonalisation (DP) and reduced personal accomplishment (PA) that can occur among individuals who work with other people in some capacity’.^7^ Burnout has been shown to be associated with various degrees of personal dysfunction including insomnia, psychosomatic symptoms, physical exhaustion, drug and alcohol abuse, and family and relationship problems. Other consequences of burnout include deterioration in quality of care, increased turnover of staff and frequent absenteeism from work.^7^

Burnout among doctors has been a global health crisis even before the COVID-19 pandemic.^8,9^ Shanafelt et al.^9^ found that up to 50% of American physicians experience burnout, with emergency physicians, family physicians and internal medicine physicians demonstrating the most significant risk. There are fewer studies on burnout in South African doctors; however, the findings are concerning.^10,11,12,13,14,15,16,17,18^ A study by Liebenberg et al.^10^ found high levels of burnout among rural doctors working in the Western Cape (WC). Rossouw et al.^11^ found 76% of doctors to have burnout in community healthcare clinics and district hospitals in Cape Town. Similarly, both Hain et al.^12^ and Naidoo et al.^13^ found a high prevalence of burnout in doctors in KwaZulu-Natal (KZN). Zeijlemaker^14^ and Moosa’s study found the prevalence of burnout among medical registrars at the University of the Witwatersrand to be higher than described in national and international literature at 84%. Rajan^15^ and Engelbrecht’s study had similar findings, with high levels of burnout in doctors working in emergency care in the public sector hospitals in Gauteng.

Several international studies have found burnout, depression, anxiety, stress and somatic symptoms to be prevalent in HCWs during the COVID-19 pandemic.^1,2,3,4,19^ The literature suggests that burnout is exacerbated during a pandemic.^20,21,22,23^ In South Africa, Lombard et al.^24^ found unacceptably high levels of post-traumatic stress symptoms in anaesthetists during the second wave of COVID-19. There are currently no further studies on pandemic-related burnout and psychological symptoms in frontline doctors in South Africa. However, there are several studies that describe elevated stress, anxiety and depressive symptoms in doctors working in South Africa outside the context of the COVID-19 pandemic.^11,12,13^ Stress is often the driving force behind burnout, functional impairment, post-traumatic stress disorder, substance abuse, psychiatric disorders and suicide. It is crucial to identify and support frontline doctors who are not coping in the context of a pandemic.^25^

The primary objectives of the study were to determine the prevalence of burnout, psychological and somatic symptoms and the association between them, experienced by frontline doctors during the COVID-19 pandemic. The secondary objectives were the association between burnout and psychological symptoms, and different demographic data, family dynamics, exercise habits, medical and psychiatric co-morbidities, and substance use habits (cigarette smoking and alcohol consumption). We also explored suggestions by frontline doctors as to measures that may mitigate burnout and psychological distress during the COVID-19 pandemic. The purpose of this component was exploratory to gain meaningful insights from the respondents to complement our interpretation of the quantitative dataset.

## Methods

### Design

A descriptive cross-sectional observational study was conducted.

### Setting

The study was conducted in the emergency departments, COVID-19 and person under investigation (PUI) tents, wards, and intensive and high care units in nine public sector hospitals in Tshwane, Gauteng.

### Sample

The sample was obtained by inviting all eligible medical doctors working on the frontlines during the COVID-19 pandemic to complete the survey. We did not implement other sampling strategies due to practical difficulties encountered during the pandemic. The study included doctors working in the departments of family medicine, emergency medicine, internal medicine and critical care. Part-time doctors were excluded from the study as were interns with less than 1 year of work experience. It was anticipated that newly employed interns may experience higher burnout and stress due to factors other than the pandemic. Sessional doctors were anticipated to experience less burnout and psychological symptoms because they worked fewer hours during the pandemic.

### Data collection

The survey was conducted during the COVID-19 pandemic between November 2020 and June 2021. A link to an online survey, using SurveyMonkey, was shared with the doctors’ WhatsApp work group. To increase the response rate, the primary researcher attended one of the morning meetings for each of the departments to present the study and address any questions. To limit response bias, sensitising terms such as ‘burnout’, ‘depression’, ‘anxiety’, ‘stress’ and ‘somatic symptoms’ were excluded from the survey invitation.

Attempts were made to obtain the exact number of doctors working in each of the included hospitals which was unfortunately unsuccessful. The population size was determined based on an estimate of the number of doctors working at the different hospitals in COVID-19 at the time. There were two departments that did not assist or distribute the survey. The estimated population was 200 frontline doctors. Completed questionnaires were captured on the SurveyMonkey server. Approximately 50% of participants were anticipated to have burnout. A sample size of 132 was recommended to describe the sample with a confidence level of 95% and a margin of error of 5%.

To assess burnout, the Maslach Burnout Inventory (MBI) was used (Mind Garden Inc. Menlo Park, California). It includes 22 items that are self-scored by participants on a seven-point Likert scale ranging from 0 (never) to 6 (every day). It is the most frequently used instrument in the international literature for measuring burnout.^8,26^ It has also been validated in South Africa in 2004 for emergency medical technicians (including doctors).^27^ The MBI has three subscales to measure burnout syndrome: EE, DP and reduced PA.^7^ High scores for EE (≥27) and DP (≥13) and low scores for PA (≤31) were suggestive of burnout.

When using the MBI, it is imperative to note that there are significant variations in the literature as to the ways burnout levels are categorised and reported. There are differences in cutoff values of the MBI subscales. This may lead to some difficulties in comparative studies. Consensus on the diagnosis of burnout remains a priority topic for future research.^8,26^ For this study, clinical burnout was defined as high EE and/or high DP scores, and extreme burnout as high EE and DP scores, together with low PA scores.

The second validated instrument used was the Depression, Anxiety, Stress Scale-21 (DASS-21), which is freely available and widely used in the literature. It is a 21-item self-administered questionnaire with three scales, namely depression, anxiety and stress, each with seven items. The DASS-21 was validated in South Africa in 2019 in a non-clinical sample of working adults in South Africa, of which 16% of the study participants were in healthcare.^28^

Other data collected included participant demographics, specialty, designation, number of years of medical working experience, overtime worked per week, exercise habits, smoking and alcohol use habits, number of child dependents, relationship status and medical or psychiatric co-morbidities. It also included any somatic symptoms that have been experienced by participants. Finally, we incorporated a qualitative question that explored suggestions as to measures that may mitigate burnout and psychological symptoms during the pandemic. The purpose of this open-ended question was exploratory to gain insights from frontline doctors that would further complement our understanding of the quantitative dataset.

The confirmability of the qualitative component of this study may be influenced by the researchers’ biases and reflexivity. The researchers involved in interpretation of the results included a doctor and a statistician, thus someone who understands the context of the doctors working on the frontlines and someone independent who may be more objective than subjective in interpretation of the answers.

### Data analysis

#### Statistical analysis

The data from SurveyMonkey database were downloaded into an Excel spreadsheet for analysis using the Statistical Package for the Social Sciences version 22 software (SPSS 22). Quantitative data were analysed using basic descriptive statistics as well as independent sample *t*-tests and analyses of variance (ANOVA) to examine differences between groups. In addition, Pearson correlations as well as regression analyses were used to examine relationships between the various levels of burnout and depression, anxiety and stress. Statistical significance was evaluated at the *p* < 0.05 level.

#### Qualitative response analysis

Qualitative data were analysed using thematic analyses to delineate the main themes. The data stemming from the open-ended questions were coded utilising open coding and analysed. Following the initial coding process, labels were assigned to the various codes. These labels were subsequently analysed and grouped according to the main themes identified within the data. The themes from the various groups were also compared.

## Results

### Demographics

The baseline demographics of the respondents are represented in Table 1. There were 206 recorded responses. The data were cleaned in line with the inclusion and exclusion criteria, which left 187 participants who had completed the section on demographics and somatic symptoms. This resulted in a response rate of 93.5% which is higher than anticipated and is likely that we underestimated the population size. Of these 187 participants, 171 had completed the MBI and 163 had completed the MBI and DASS-21. A total of 163 responses were therefore suitable for statistical analysis, with 119 females (73.0%) and 44 males (27%). Most of the respondents were between the ages of 25 and 34 years (*n* = 102; 62.6%) with a mean age of 33.71 years. Almost half of the sample worked in family medicine (*n* = 80; 42.8%) and the most common designation was that of a medical officer (*n* = 57; 30.5%). A total of 125 (66.8%) of the respondents work between 13 h and 20 h overtime every week and most of their work areas involved COVID-19/PUI tents (*n* = 88; 54%) and the emergency unit (*n* = 96; 58.9%). Just over half of the sample (*n* = 86; 52.8%) have between 1 and 5 years of work experience, with an overall sample average of 7 years of work experience. Most respondents did not have child dependents (*n* = 98; 60.1%). We found 19% of our sample to have a comorbid medical condition and 15.3% to have an underlying psychiatric condition.

**TABLE 1:** Demographics of respondents.

Variable	*n*	%	Mean	Standard deviation	Min.	Max.	Range
**Gender**							
Female	119	73.0	-	-	-	-	-
Male	44	27.0	-	-	-	-	-
**Relationship status**							
Single	27	16.6	-	-	-	-	-
In a relationship	33	20.2	-	-	-	-	-
Married	99	60.7	-	-	-	-	-
Divorced	4	2.5	-	-	-	-	-
Widowed	0	0.0	-	-	-	-	-
**Number of dependents**							
None	98	60.1	-	-	-	-	-
1	24	14.7	-	-	-	-	-
2	27	16.6	-	-	-	-	-
3 or more	14	8.6	-	-	-	-	-
**Age group (years)**							
25–34	102	62.6	-	-	-	-	-
35–44	44	27.0	-	-	-	-	-
45–54	13	8.0	-	-	-	-	-
55–64	3	1.8	-	-	-	-	-
65+	1	0.6	-	-	-	-	-
**Age (years)**	-	-	33.710	7.927	25	68	43
**Speciality**							
Family medicine	75	46.0	-	-	-	-	-
Emergency medicine	30	18.4	-	-	-	-	-
Internal medicine	31	19.0	-	-	-	-	-
Critical care	3	1.8	-	-	-	-	-
Other	24	14.7	-	-	-	-	-
**Designation**							
Intern	31	19.0	-	-	-	-	-
Community service medical officer	27	16.6	-	-	-	-	-
Medical officer	48	29.4	-	-	-	-	-
Registrar	29	17.8	-	-	-	-	-
Specialist	28	17.2	-	-	-	-	-
**Overtime**							
None	7	4.3	-	-	-	-	-
4 h – 8 h	4	2.5	-	-	-	-	-
9 h – 12 h	11	6.7	-	-	-	-	-
13 h – 20 h	107	65.6	-	-	-	-	-
> 20 h	34	20.9	-	-	-	-	-
**Areas of work during past 8 months**							
COVID-19/PUI tents	88	54.0	-	-	-	-	-
COVID-19/PUI wards	66	40.5	-	-	-	-	-
Emergency unit	96	58.9	-	-	-	-	-
COVID-19/PUI intensive or high care units	35	21.5	-	-	-	-	-
Internal medicine	46	28.2	-	-	-	-	-
Other	45	27.6	-	-	-	-	-
**Work experience since qualification**							
1–5	86	52.8	-	-	-	-	-
6–10	32	19.6	-	-	-	-	-
11–15	18	11.0	-	-	-	-	-
16–20	10	6.1	-	-	-	-	-
> 20	17	10.4	-	-	-	-	-
**Years of experience**	-	-	7.035	6.897	1	45	-
**Comorbid medical conditions**							
Yes	31	19.0	-	-	-	-	-
No	132	81.0	-	-	-	-	-
**Psychiatric conditions**							
Yes	25	15.3	-	-	-	-	-
No	138	84.7	-	-	-	-	-
**Consume alcohol**							
Yes	104	63.8	-	-	-	-	-
No	59	36.2	-	-	-	-	-
**Smoke tobacco**							
Yes	15	9.2	-	-	-	-	-
No	148	90.8	-	-	-	-	-

PUI, person under investigation; COVID-19, coronavirus disease 2019; Min., minimum; Max., maximum.

### Burnout

Most of the sample scored high on EE (*n* = 89; 54.6%) and DP (*n* = 58; 35.6%), with 64 (39.3%) scoring low on PA (Table 2). All three scales of burnout (EE, DP and PA) have good internal consistency, with Cronbach’s alpha coefficients of 0.92, 0.77 and 0.81, respectively. Moderate to high EE was present in 132 (81%), moderate to high DP in 110 (67.5%), and moderate to low PA in 116 (71.2%) doctors. Table 3 shows the cross-tabulation results of all three components of burnout. Of our sample, 96 (58.9%) doctors had clinical burnout (either high EE or DP scores) and 32 (19.6%) had extreme burnout (high EE, high DP together with low PA scores). Furthermore, 114 (69.9%) had either high EE or DP or low PA scores.

**TABLE 2:** Frequencies of emotional exhaustion, depersonalisation and personal accomplishment.

Frequency	*n*	%
**Emotional exhaustion**		
Low	31	19.0
Moderate	43	26.4
High	89	54.6†
**Depersonalisation**		
Low	53	32.5
Moderate	52	31.9
High	58	35.6†
**Personal accomplishment**		
Low	64	39.3†
Moderate	52	31.9
High	47	28.8

† , Highest response frequency.

**TABLE 3:** Burnout prevalence.

Emotional exhaustion	Depersonalisation	Personal accomplishment		
		Low	Moderate	High
Low	Low	2	5	16
	Moderate	2	2	3
	High	0	1	0
Moderate	Low	3	5	8
	Moderate	11	5	5
	High	2	2	2
High	Low	3	7	4
	Moderate	9	10	5
	High	32†	15	4

† , Highest response frequency.

### Psychological symptoms

**FIGURE 1:**
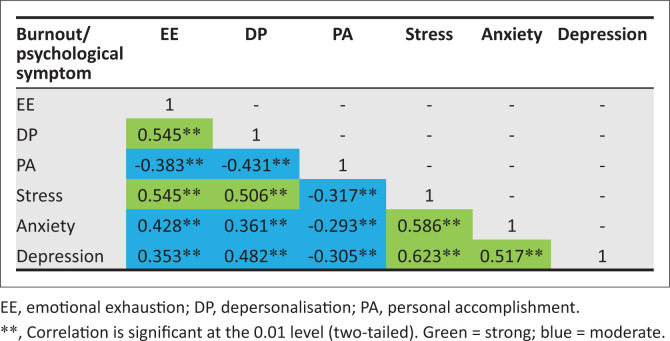
Correlation between burnout and psychological symptoms.

Our sample showed moderate to extremely severe levels of the following psychological symptoms: stress in 90 (55.1%), anxiety in 71 (43.6%) and depression in 36 (22.1%) participants (Table 4). Any symptoms of stress, anxiety or depression (mild to extremely severe) were present in 118 (72.4%), 84 (51.5%) and 55 (33.7%) doctors, respectively. All three scales (stress, anxiety and depression) that compromise psychological symptoms have good internal consistency, with Cronbach’s alpha coefficients 0.88, 0.77 and 0.92, respectively. Figure 1 shows there is significant correlation between all burnout (EE, DP and PA) and all the psychological symptoms (depression, anxiety and stress).

**TABLE 4:** Frequencies and prevalence of psychological symptoms.

Frequency	*n*	%
**Stress**		
Normal	45	27.6
Mild	28	17.2
Moderate	54	33.1†
Severe	18	11.0†
Extremely Severe	18	11.0†
**Anxiety**		
Normal	79	48.5
Mild	13	8.0
Moderate	38	23.3†
Severe	19	11.7†
Extremely Severe	14	8.6†
**Depression**		
Normal	108	66.3
Mild	19	11.7
Moderate	16	9.8†
Severe	12	7.4†
Extremely Severe	8	4.9†

† , Moderate to extremely severe symptoms.

### Somatic symptoms

It was found that the sample of doctors experienced adverse somatic symptoms (Table 5). Fatigue was the most prevalent somatic symptom. A Kruskal–Wallis test showed that there was a statistically significant difference in EE, DP, low PA, depression, anxiety and stress between the experiences of the listed somatic symptoms. Post-hoc analyses revealed that those that rated their experience of certain somatic symptoms as ‘moderate’ and ‘high’ recorded a higher median score than those who rated their experience as ‘low’.

**TABLE 5:** Somatic symptom prevalence.

Somatic symptom	Never (0)		Rarely (1)		Sometimes (2)		Often (3)		Always (4)		Mean	Standard deviation
	*n*	%	*n*	%	*n*	%	*N*	%	*n*	%		
Headaches	22	13.5	43	26.4	61	37.4†	36	22.1	1	0.6	1.699	0.982
Fatigue	5	3.1	7	4.3	48	29.4	76	46.6†	27	16.6	2.693	0.905
Muscle Tension	29	17.8	23	14.1	55	33.7†	42	25.8	14	8.6	1.933	1.208
Insomnia	42	25.8	36	22.1	57	35.0†	19	11.7	9	5.5	1.491	1.157
Nightmares	80	49.1†	50	30.7	28	17.2	5	3.1	0	0.0	0.742	0.850
Epigastric Pain	84	51.5†	29	17.8	29	17.8	19	11.7	2	1.2	0.933	1.128
Abdominal Pain	95	58.3†	29	17.8	31	19.0	6	3.7	2	1.2	0.718	0.978
GIT Upset	55	33.7†	45	27.6	48	29.4	13	8.0	2	1.2	1.153	1.022
Palpitations	82	50.3†	34	20.9	29	17.8	17	10.4	1	0.6	0.902	1.073
Irritability	20	12.3	24	14.7	62	38.0†	46	28.2	11	6.7	2.025	1.094
Nervous Breakdown	97	59.5†	33	20.2	25	15.3	7	4.3	1	0.6	0.663	0.931
Chest Discomfort	106	65.0†	33	20.2	21	12.9	2	1.2	1	0.6	0.522	0.811
Change in Dietary Habits	43	26.4	25	15.3	51	31.3†	37	22.7	7	4.3	1.632	1.217
Over-reacting to situations	28	17.2	36	22.1	60	36.8†	32	19.6	7	4.3	1.718	1.097

† , Highest response frequency.

### Respondent characteristics

The relationship between burnout and psychological symptoms, and age was investigated using Pearson-product moment correlation coefficient. There was a significant, small, negative correlation between age and DP, *r* = −0.177, *p* < 0.05, with an increase in age associated with a lower DP score. We found a significant, moderate, negative correlation between age and depression, *r* = −0.160, *p* < 0.05, with an increase in age associated with lower depression.

Analyses of variance showed that there was a statistically significant difference at *p* < 0.05 in DP for the five different designations: *F* (4) = 2.643, *p* = 0.036. Post-hoc comparisons indicated that the mean score for an intern (x = 12.355, standard deviation [s.d.] = 7.069) was significantly different from a registrar (x = 8.759, s.d. = 6.833) and specialist (x = 8.250, s.d. = 7.594). The mean score for a medical officer (x = 11.854, s.d. = 6.435) was also statistically different from a specialist (x = 8.250, s.d. = 7.594). We found no statistically significant differences between psychological symptoms and designation.

No statistically significant differences between burnout or psychological symptoms and gender, specialities, or duration of work experience were found. Furthermore, no statistically significant differences in medical or psychiatric co-morbidities and burnout or psychological symptoms were found.

### Family dynamics

There was a statistically significant difference at *p* < 0.05 in DP (*F* (4) = 3.454, *p* = 0.018) for respondents with child dependents. Regarding burnout (DP specifically), post-hoc comparisons indicated that the mean score for individuals with no or zero dependents (x = 11.98, s.d. = 7.36) was significantly different from those with two (x = 7.37, s.d. = 6.22) and three or more dependents (x = 7.57, s.d. = 4.36).

Analysis of variance (ANOVA) showed that there was a statistically significant difference at *p* < 0.05 in stress (*F* (4) = 2.868, *p* = 0.038) and depression (*F* (4) = 3.143, *p* = 0.027) for respondents with child dependents. Firstly, in relation to stress, post-hoc comparisons indicated that the mean score for individuals with no or zero dependents (x = 16.18, s.d. = 9.38) was significantly different from those with two (x = 11.93, s.d. = 7.37) and three or more dependents (x = 10.86, s.d. = 5.42). Secondly, depression was found to differ for those who had no dependents (x = 13.55, s.d. = 10.76) and two (x = 8.15, s.d. = 6.42) as well as three dependents (x = 8, s.d. = 6.93).

No statistically significant differences in burnout or psychological symptoms in relation to relationship status were found.

### Substance use and exercise habits

Findings showed that almost two-thirds of respondents (*n* = 104; 63.8%) consume alcohol, and 15 were smokers (9.2%). Of the participants who consumed alcohol, 57 (54.8%) had the same alcohol consumption habits as before the pandemic and 17 (16.3%) consumed more alcohol. Of those who smoked, eight (53.3%) smoked more than before the pandemic. Of the sample, 30 respondents (18.4%) never exercise, and the highest frequency of exercise was one to three times per week (*n* = 100; 61.3%).

No statistically significant differences were found between exercise and burnout or psychological symptoms. However, statistically significant differences were found between various somatic symptoms and exercise habits, with increased frequency of exercising associated with fewer somatic symptoms. Findings showed no statistically significant differences between substance use habits and burnout or psychological symptoms.

### Suggested measures to mitigate burnout and psychological symptoms

The qualitative component of the questionnaire was coded and analysed by two researchers to ensure veracity of the findings. Of the 163 participants included in the study, only 151 participants answered this question. Main themes emerged as potential measures of combatting burnout and psychological distress during the pandemic. These included the need for more resources such as equipment, better working conditions, support from management, financial compensation, psychological support and finally measures to promote healthy living. No new codes emerged, and thematic saturation was obtained.

## Discussion

Our study highlights a concerning prevalence of burnout, psychological and somatic symptoms in doctors working on the frontlines during the COVID-19 pandemic in public sector hospitals in Tshwane. Findings showed that 54.6% of doctors had high levels of EE. Most doctors working on the frontline in this study felt emotionally overextended and depleted by their work. Furthermore, 35.6% of doctors had high levels of DP. These doctors felt cynical, negative, hostile and excessively detached from their work. Low PA was present in 39.3% of doctors. These doctors had a growing sense of failure in their ability to do their job well. They experienced feelings of inadequacy, incompetency and non-productivity.^8^ Findings showed 69.9% of doctors to have high EE, high DP or low PA. Clinical burnout was present in 58.9% and extreme burnout in 19.6% of doctors. A systematic review conducted by Ghahramani et al.^22^ on burnout among HCWs during the COVID-19 pandemic reported the overall prevalence of burnout to be 52% (95% confidence interval [CI]: 40% – 63%) among all HCWs. This is higher than rates reported in other pandemic-related studies conducted over the past two decades^2,3,22^, but lower than found by our study. Furthermore, our study showed that burnout, especially higher EE, or DP was associated with the experience of adverse somatic symptoms. This is similar to a study conducted by Barello et al.^19^ during the pandemic peak in Italy.

Moderate to severe stress, anxiety and depression were present in 55.2%, 43.6% and 22.1% of participants, respectively. Considering the fact that the lifetime prevalence of depression and anxiety has been documented as 9.8% and 15.8%, respectively, in the general population of South Africa, these findings are worrisome.^29^ Findings from this study were compared to several systematic reviews on pandemic-related anxiety and depression in HCWs. Pappa et al.^4^ reported a similar prevalence of depression in 22.8%, but a much lower prevalence of anxiety in 23.2% of HCWs compared to our study. Elevated stress among frontline doctors in our study was comparable to Busch et al.^2^ but higher than Salazar et al.^3^ who reported a prevalence of 56.8% and 37.8%, respectively.

Table 6 shows comparisons in burnout, depression and anxiety in public sector doctors found by different studies conducted in South Africa. Regarding burnout and moderate to severe depression, it should be noted that our study has comparable findings to that of Naidoo et al.^13^ which investigated burnout in medical doctors in KZN public sector hospitals. Findings from this study showed a remarkably similar prevalence of burnout to the study of Coetzee^16^ and Kluyts, which investigated burnout among anaesthetists in the public sector in South Africa in 2018. A study by Hain et al.,^12^ which was also conducted during the COVID-19 pandemic, found higher levels of depression in doctors working in rural KZN than our study. Similarly, Rossouw et al.^11^ found higher levels of depression in doctors working in WC community healthcare clinics and district hospitals. Our study, however, found much higher levels of anxiety than both Hain et al.^12^ and Naidoo et al.^13^ (approximately double prevalence of anxiety).

**TABLE 6:** Comparisons in burnout subscales, depression and anxiety between recent studies in South African doctors working in the public sector.

Study	Current study (COVID-19, Tshwane)	Hain et al.^12^	Liebenberg et al.^10^	Naidoo et al.^13^	Rossouw et al.^11^	Rajan and Engelbrecht^15^	Zeijlemaker and Moosa^14^	Van der Walt et al.^17^	Coetzee and Kluyts^16^
Year (sample size)	2021 (*n* =163)	2020 (*n* = 96)	2013 (*n* =36)	2019 (*n* = 150)	2010 (*n* = 132)	2016 (*n* = 93)	2018 (*n* = 170)	2013 (*n* = 124)	2018 (*n* = 498)
High EE%	54.6	58.4	56	48.7	53	66.7	66.5	45.2	49
High DP%	35.6	59.6	75	45.3	64	53.8	74.7	50	38
Low PA%	39.3	48.3	44	43.3	43	22.6	52.4	46	41
Moderate-high EE%	81	77.5	89	70	78	92.5	85.9	72.6	70
Moderate-high DP%	67.5	76.5	89	67.3	87	80.7	94.1	78.2	66
Moderate-low PA%	71.2	73	86	78	74	69.9	77.7	81.5	70
Clinical burnout†%	58.9	68.5	81	58.7	76	-	84	-	-
Extreme burnout‡%	19.6	-	31	-	-	-	-	21	17.5
High scores in either three subscales %	69.9	-	-	-	84	-	-	-	-
Moderate-severe Depression %	22.1	35.6	-	21	30	-	-	-	-
Moderate-severe anxiety %	43.6	23.3	-	20	-	-	-	-	-

EE, emotional exhaustion; DP, depersonalisation; PA, personal accomplishment.

† , Clinical burnout: high EE and or high DP.

‡ , Extreme burnout: high EE, high DP and low PA.

It is interesting to note that percentages pertaining to the EE and PA subscales are relatively similar across studies in South Africa, except perhaps for studies conducted by Rajan^15^ and Engelbrecht and Zeijlemaker^14^ and Moosa which both had higher EE levels. Rajan^15^ and Engelbrecht’s study investigated burnout in doctors working in emergency medicine across Gauteng, whereas Zeijlmaker^14^ and Moosa’s study investigated burnout in registrars working in the Witwatersrand circuit. Both studies were conducted in academic centres in Gauteng where there may have been higher academic expectations on doctors contributing to higher EE. In contrast to similar EE and PA percentages between studies, it is enlightening to see great variability between studies in DP levels. Higher DP levels were found in doctors working in rural KZN, rural WC, WC community health clinics and district hospitals, Gauteng emergency units, and registrars in the Witwatersrand circuit.^10,11,12,14,15^ Individual and situational factors that influence DP in doctors in South Africa may warrant further investigation, as the great risk of DP, in addition to burnout, is the development of dehumanisation which compromises patient care.

International literature suggests that burnout is exacerbated during a pandemic. Our study, however, did not demonstrate higher levels of burnout during the COVID-19 pandemic. The reasons for this are likely multifactorial. The hard lockdown and alcohol ban implemented in South Africa during the pandemic reduced the volume of trauma cases, which is usually a great burden to our public hospitals. These findings may also not be generalisable to other settings such as rural hospitals, other provinces or the private sector. The data collected during this study were mostly at the beginning of the pandemic which may have influenced results, as burnout may take some time to develop. A study conducted by Mercuri et al.^20^ found less than 20% of Canadian EU physicians had burnout during the first wave of the COVID-19 pandemic. During the second wave, burnout prevalence had increased to 60%. Lastly, it has been argued that doctors working in resource-limited countries are accustomed to resource shortages and facing adversity which develops resilience, which in turn is protective against burnout.^22,30^

Findings showed a significant correlation between all burnout components and all psychological symptoms. Stress in particular was found to be strongly associated with higher EE and DP, which supports the assertion that chronic stress is the driving force behind burnout. Furthermore, stress was strongly associated with both anxiety and depression, and depression was strongly associated with anxiety. This highlights the devastating consequences of elevated stress, which not only drives burnout but also psychological disorders and somatic symptoms. In light of this, the raised stress levels (72.4%) in our cohort of doctors are highly concerning. Other studies have also shown burnout to be associated with anxiety and depression.^12,31^

Interestingly, both this study and Caliskan^32^ and Dost’s study did not find individuals with underlying psychiatric or medical conditions to be more predisposed to depression, anxiety or stress. Furthermore, findings also showed that they were not at increased risk for burnout, although they may have been more at risk for certain somatic symptoms, similar to a study by Chew et al.^1^ This insinuates that even the mentally and physically healthy doctor is at risk of burnout and psychological repercussions.

No differences in burnout or psychological symptoms between genders were found. Although numerous other studies have found burnout (especially EE), as well as anxiety, depression and stress, to be higher in females.^4,12,13,19,20,33,34,35,36,37^ Similar to other studies, this study showed older age was associated with less DP and depression.^15,20,33,35^ Regarding relationship status, no difference in burnout or psychological symptoms was found. This contrasts Peltzer et al.^18^ who found marriage to be protective against burnout and Suryavanshi et al.^38^ and Elbay et al.^33^ who found single participants at greater risk of mental health issues. Like Mercuri et al.,^20^ this study showed that having child dependents was associated with less DP, thus, protective against burnout.

### Study limitations

Owing to the cross-sectional nature of the study, causal relationships may not be reliably determined. The great variability between different health institutions and provinces in South Africa makes generalizability of the study difficult. Another limitation of this study is that no specific sampling method was implemented, but rather all eligible doctors were invited to partake in the study. Pandemic-related challenges such as COVID-19 restrictions on meetings, as well as the shift-work nature of medicine made sampling strategies practically difficult. This may have introduced self-selection bias and response bias. It is possible that doctors with higher burnout scores may have declined to participate, and the prevalence of burnout has been underestimated in this study. Another point to consider is that it is difficult to make a clear conclusion regarding increased burnout based on a single cross-sectional study as there are no comparatives. Although attempts were made to determine the exact population size, this was unsuccessful, and the population determined based on an estimate. Based on our unusually high response rate of 93.5%, it likely that we underestimated the population size. We do not, however, believe this would have significantly impacted the results. A global systematic review by Meyer et al.^39^ on response rates in patient and healthcare professional surveys in surgery found the average response rates in over 1746 healthcare professionals’ surveys to be 53.3% ± 24.5%. Furthermore, they found that in-person surveys yielded higher response rates than online surveys. Our study incorporated both online and in-person surveys which likely increased the response rate. If we estimate that our response rate was more realistically around 65%, this would have meant that our population size was closer to 288 and the sample size needed to describe the sample with a confidence level of 95% and a margin of error of 5% would have needed to be 165. This is still close to the sample size in this study. Nonetheless, a larger sample size would have provided more insight, and the unusually high response rate should be interpreted with caution and may influence generalizability of the study. The overlap of this study from November 2020 to June 2021 may have resulted in a larger population than initially anticipated as new appointments are frequently made in the beginning of the year. This was unavoidable due to unforeseen delays in the ethical process. The qualitative component of the study was limited and more exploratory in nature rather than an in-depth analysis and is consequently less impactful. The anonymity of the study may have made it easier for respondents to be more honest than if they were partaking in an interview where they may know the researchers. However, further exploration, clarification and in-depth analysis were not possible using this method of data collection. A final limitation was that of missing data in the dataset which could lead to a loss of information.

## Conclusion

This study highlights the calamitous psychophysical consequences of the COVID-19 pandemic on frontline doctors in South Africa. Elevated burnout, anxiety, depression, stress and adverse somatic symptoms were found in our cohort of doctors. The prevalence of stress and anxiety was higher than that reported in the national and international literature. The study showed a significant correlation between all burnout and all psychological symptoms. It was not found that the pandemic caused worsening burnout as the international literature suggests; however, it is difficult to make a clear conclusion regarding worsening burnout based on a single cross-sectional study as there are no comparatives. Undeniably, the severe level of burnout in doctors working in South Africa deserves priority attention. Measures suggested by frontline doctors to mitigate psychological strain included the need for more resources such as equipment, better working conditions, support from management, financial compensation, psychological support and finally measures to promote healthy living. These suggestions should be considered by policy-makers in the current climate as well as in the event of future pandemics. Further research is needed to establish evidence-based interventions to alleviate vulnerability, strengthen resilience, improve organisational shortcomings, and safeguard the mental and physical health of frontline doctors, especially under pandemic conditions.
